# Concurrent Use of Cigarettes and Smokeless Tobacco among US Males and Females

**DOI:** 10.1155/2012/984561

**Published:** 2012-05-16

**Authors:** Nasir Mushtaq, Mary B. Williams, Laura A. Beebe

**Affiliations:** Department of Biostatistics and Epidemiology, University of Oklahoma Health Sciences Center, 801 NE 13th Street, CHB-309, Oklahoma City, OK 73104, USA

## Abstract

*Background*. The current study describes concurrent use of cigarettes and smokeless tobacco (CiST) among males and females and evaluates factors associated with CiST use. *Methods*. Cross-sectional data were drawn from the 2010 Behavioral Risk Factor Surveillance System (BRFSS). Weighted stratified analyses were performed to find associations between CiST use and sociodemographic factors by gender. CiST users were compared to three different tobacco use groups: nonusers, exclusive smokers, and exclusive ST users. *Results*. Younger age and heavy alcohol consumption were consistently associated with increased odds of CiST use among both males and females, and regardless of comparison group. Among males, education was inversely related to CiST use, and these findings were consistent in all three comparisons. Among women, those unable to work or out of work were more likely to be CiST users, which was consistent across comparisons. American Indian females had higher odds of CiST use than White females when nontobacco users or smokers were the comparison group. *Conclusion*. This study identified sociodemographic characteristics associated with CiST use, and differences in these associations among women and men. Additionally, this study highlights the need to carefully consider what comparison groups should be used to examine factors associated with CiST use.

## 1. Introduction

Tobacco use is widely considered the most preventable cause of illness and death in the United States. Although the consumption of cigarettes and some other forms of tobacco have decreased in the last decade [1, 2], the consumption of smokeless tobacco has recently increased [3]. In addition, traditional cigarette companies, such as Reynolds America and Altria, the parent company of Phillip Morris, have extended their product lines to include many types of smokeless tobacco [4]. Not only are tobacco companies moving into the smokeless tobacco market, they are marketing smokeless tobacco products as alternatives to smoking when there are bans or restrictions [5]. These conditions encourage the dual use of cigarettes and smokeless tobacco.

The combined use of any tobacco products may increase exposure to potentially harmful chemicals and subsequently increase risk of disease [6, 7]; however, evaluating the concurrent use of cigarettes and smokeless tobacco (ST) is especially important for four reasons. First, tobacco marketing of smokeless tobacco as an alternative when smoking is restricted may increase the prevalence of concurrent use. Second, these two forms of tobacco are the most prevalent forms of tobacco used, and a higher proportion of both groups use tobacco daily compared to users of other forms of tobacco [8, 9]. Third, increased health risks of concurrent use have been demonstrated, such as, an increased risk of acute myocardial infarction among concurrent users beyond the risk of only smoking or solely using smokeless tobacco [7]. Finally, concurrent users may be less likely than cigarette smokers to report intentions to quit in the next 6 months [10].

Although concurrent tobacco use has been previously examined in specific populations in the United States since 1999 [11–15], there is limited research describing concurrent use in the general US adult population. Prevalence of concurrent use among men did not significantly change from 1992 (1.0% 95% CI: 1.0–1.1) to 2002 (0.9% 95% CI: 0.8–1.0) [16, 17]. However, the most recent Federal Trade Commission report on smokeless tobacco (ST) found that snuff sales have recently risen [3]. The increased ST sales may reflect an increased uptake of ST by cigarette smokers, especially in light of tobacco companies' marketing ST products to smokers [5]. Recent studies have reported higher prevalence of concurrent use from national surveys. One study utilizing Behavioral Risk Factor Surveillance Survey (BRFSS) from 2008 for selected states reported a prevalence of concurrent use of 1.5% [18], while a nationwide consumer-based survey found that an overall prevalence of concurrent use was 1.1% [10]. Another recent study of 2009 BRFSS data found that concurrent use ranged by state from 0.9% in Puerto Rico to 13.7% in Wyoming and differed among men and women [19].

Other studies have evaluated ST use among cigarette smokers and cigarette smoking among ST users, which can provide important information given the changing patterns in tobacco use. One such study reported that 6.1% of adult smokers used ST, and 41.3% of ST users smoked cigarettes [10]. Furthermore, Tomar and colleagues found among men 2.3% of daily smokers and 4.3% of someday smokers also used snuff, while 15% of daily snuff users and 45% of someday snuff users also smoked cigarettes [4].

A limited number of studies have examined correlates of concurrent tobacco use. The consumer-based study found that prevalence of concurrent use was higher among young, men, lower income (<$15,000), and White respondents [10]. A study of Air Force recruits found that ST use among smokers was associated with age (17–20 years), sex (males), race (Whites), and alcohol consumption (at least once per week) [20]. In a similar recent study, concurrent use among active duty military personnel found factors associated with a higher prevalence of concurrent use compared to nontobacco use included: male gender, younger age (21–34 years old), less than a college education, and not being married [21].

These studies highlight the need for ongoing surveillance of concurrent use, and although some have provided information regarding the prevalence of concurrent tobacco use in different populations, questions remain regarding factors associated with concurrent use among women. To increase our understanding of the concurrent use of cigarettes and ST (CiST) in various groups, the current study examined CiST prevalence and factors associated with CiST use by gender. Furthermore, questions remain regarding the appropriate comparison group for CiST users. Most previous studies have compared concurrent users to cigarette smokers and/or smokeless tobacco users; [4, 10, 20, 21] however, it may be of interest to also compare CiST users to nontobacco users (nonusers). Therefore, CiST users were compared to: exclusive smokers, exclusive ST users, and nonusers.

## 2. Methods and Materials

Cross-sectional data were drawn from the Behavioral Risk Factor Surveillance System (BRFSS) survey for the year 2010. Centers for Disease Control and Prevention (CDC) in collaboration with state health departments conduct BRFSS to obtain state-level data related to various behavioral risk factors, sociodemographic characteristics, and health conditions. BRFSS employs telephone interviews by random digit dialing to collect information from noninstitutionalized residents18 years and older. When combined across states, BRFSS data provide national estimates which are comparable to those obtained from other national surveys [22–24]. The ability of BRFSS to provide valid national estimates and across state comparisons is well established [25]. A number of studies in the past have used BRFSS data to study different behavioral risk factors including smoking at national level.

### 2.1. Measures

#### 2.1.1. Tobacco Use

Tobacco use status was categorized into four categories: exclusive cigarette smoking, exclusive smokeless tobacco (ST) use, concurrent use of cigarettes and ST (CiST), and no current tobacco use. Cigarette smoking was defined as respondents who smoked at least 100 cigarettes in their lifetime and currently smoke cigarettes. Exclusive smokers were those who smoked cigarettes someday or everyday and did not currently use ST. Respondents currently using ST products, someday or everyday but not currently smokers, were defined as exclusive ST users. Nontobacco users were those who were not current cigarette smokers or ST users.

#### 2.1.2. Concurrent Cigarette and Smokeless Tobacco (CiST) Use

The outcome variable for this study, CiST use, was characterized as the use of both ST and cigarettes irrespective of the frequency of use. Therefore, both daily and someday users of ST products and cigarettes were considered CiST users.

#### 2.1.3. Sociodemographic Factors

These variables included age, gender, race/ethnicity, education level, income level, occupation, marital status, and alcohol consumption. Age was categorized as 18–24, 25–34, 35–44, 45–54, 55–64, and 65 years or older.; race/ethnicity was divided into six categories, non-Hispanic white, non-Hispanic African American, non-Hispanic American Indian or Alaska Native, Hispanic, multiracial, and other. Education had four levels, less than high school, high school, some college, and college graduate or more. Participants were assigned into the following occupational categories: employed for wages, self-employed, homemaker, out of work, student, retired, and unable to work. Annual household income was categorized as less than $10,000, $10,000 to $14,999, $15,000 to $19,999, $20,000 to $24,999, $25,000 to $34,999, $35,000 to $49,999, $50,000 to $74,999, and more than $75,000. Marital status was divided into two categories: married (i.e., married and member of an unmarried couple) and single (i.e., divorced, widowed, separated, and never married). Alcohol use is a social factors routinely associated with tobacco use [26, 27]. Alcohol consumption was divided into two categories heavy drinking and no low or moderate drinking. Heavy drinking was defined by BRFSS as more than two drinks per day for men and more than one drink per day for women.

### 2.2. Statistical Analysis

**Figure 1:**
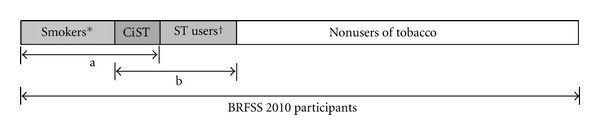
Analysis framework for different comparison groups. Comparisons: (1) CiST versus nonusers of tobacco, (2) within subgroup “a” (CiST versus exclusive smokers), and (3) within subgroup “b” (CiST versus exclusive ST users). *Exclusive smokers (daily or someday), ^†^exclusive ST users (daily or someday), CiST: concurrent users of cigarettes, and ST a: all smokers (exclusive and dual users) and b: all ST users (exclusive and dual users).

Descriptive statistics were calculated for the variables in the study. Gender stratified weighted prevalences were calculated for all the variables including tobacco use patterns and sociodemographic characteristics. Weighted stratified analyses were performed to examine associations between CiST use and sociodemographic factors by gender. CiST users were compared to three different tobacco use groups: nonusers, exclusive smokers, and exclusive ST users (Figure 1).

Chi-square goodness of fit tests and logistic regression models were used to determine bivariate associations between CiST use and sociodemographic variables. The variables found to be associated at a significance level of 0.05 with CiST use from simple logistic regression models were used in multivariate regression analysis. Adjusted odds ratios (ORs) and 95% confidence intervals were calculated as the measure of association. All analyses were conducted using SAS v 9.2 and “FINALWT” variable, recommended by BRFSS, was used as a weighting variable. Weighted analyses addressed any imbalances in the sampling design and also provided the unbiased estimates for the general population. An alpha level of 0.05 was used for statistical significance.

## 3. Results

The prevalence of CiST use was higher among males (1.6%) compared to females (0.3%). The majority of male CiST users were non-Hispanic Whites (79%), employed for wages (54%), and had some college or less education (87%). Similarly, most female CiST users were non-Hispanic Whites (73%) and attained some college or less education (84%); however, 38% of female CiST users were employed for wages. A higher proportion of male CiST users (64%) than female CiST users (47%) had an annual income more than $25,000. Sociodemographic characteristics of the participants are described in Table 1.

### 3.1. CiST Use among Males

Among men CiST use was reported by 1.6% of participants, while more than 17.4% were exclusive smokers, and 4.2% were exclusive ST users. The prevalence of CiST use among men was higher among American Indian/Alaska Natives, those reporting multiple races, less than 35 years old, those out of work or unable to work, had a high school education or less, had less income, and were single and heavy drinkers. Sociodemographic characteristics of male respondents are described in Table 2.

The multivariate logistic regression analyses using male nontobacco users as the comparison was conducted to obtain association of individual sociodemographic variable with CiST use while controlling for all other variables.  Table 3 summarizes the associations between sociodemographic factors and CiST use among males compared to exclusive smokers, exclusive ST users, and nonusers. The likelihood of CiST use increased as age decreased among men. Native American men and those reporting multiple races were about 20% more likely to be CiST users compared to white men. As educational attainment decreased the odds of being a CiST user increased with men having less than a high school education being more than seven times as likely as those with a college degree to be a CiST user. Similarly, as men's household incomes rose above $20,000, the odds of CiST use decreased. Men with incomes between $10,000 and 14,999 were also less likely to be CiST users than those making less than $10,000. Men who were out of work or were unable to work were more likely to be CiST users than men employed for wages. On the other hand, men who were self-employed, students, homemakers, or retired were less likely to be CiST users than men employed for wages. Men who also drank heavily were more than four times as likely to be CiST users as men who drank less than two drinks per day.

#### 3.1.1. Smokeless Tobacco Use among Male Smokers

CiST use was reported by 8.5% of male smokers. Results of the multiple logistic regression models comparing CiST use to exclusive smokers indicated that CiST use was higher among Whites than any other racial/ethnic group. White smokers were 1.3 times more likely to be CiST users than American Indian/Alaska Native smokers, and 2.5 times more likely than African American male smokers after adjustment. There was an inverse association between CiST use and education attained among male smokers. Compared to those who were employed for wages, other occupations were less likely to be CiST users. Similarly, heavy alcohol use increased the odds of CiST use by 1.2 times among male smokers.

#### 3.1.2. Cigarette Smoking among Male ST Users

Twenty-eight percent of the male ST users also smoked cigarettes. Male ST users who were White were less likely to also smoke cigarettes compared to any other race/ethnic group, except American Indian/Alaska Native ST users. Men who graduated from college were less likely to be CiST users compared to those with some college or high school education, and less than half as likely to be CiST users than those with less than high school education. CiST use was also higher among male ST users with annual incomes less than $10,000 compared to those earning more than $10,000. CiST use among male ST users was 1.7 times higher among heavy drinkers compared to nonheavy alcohol drinkers.

### 3.2. CiST Use among Females

CiST use was reported by 0.3% of the female participants, while 14.8% were exclusive smokers, and 0.5% were exclusive ST users. AI/AN women had the highest prevalence of CiST use (1.5%) and smoking (29.8%). Like men, CiST use among women increased with decreasing education level. Similarly, CiST prevalence among women decreased with increasing income level. A higher proportion of women who were unable to work were CiST users (1.1%) followed by those who were out of work (0.7%). Sociodemographic characteristics of the female respondents stratified by their tobacco use status are summarized in Table 4.

Results of multivariate logistic regression analyses (Table 5) using female nontobacco users as the comparison indicated after controlling for all other variables, AI/AN females were almost twice likely to be CiST users compared to White women. The likelihood of women being CiST users compared to nonusers increased as education level decreased. Women with less than a high school education were more than four times as likely to be CiST users as women with a college education. Similarly, women who were unable to work were almost three times as likely to be CiST users as those employed for wages, and those out of work were 75% more likely to use both products. As the household income of women rose above $20,000, the odds of CiST use decreased compared to those with incomes less than $10,000. Heavy alcohol drinking was associated with more than four times the odds of CiST use among women after adjustment for other covariates.

#### 3.2.1. Smokeless Tobacco Use among Female Smokers

CiST use was reported by 2.3% of the female smokers. Age, race/ethnicity, education level, income level, occupation, and heavy alcohol consumption were significantly associated with CiST use among female smokers. Among female smokers, AI/AN were 1.6 times more likely to be CiST users than White, and Hispanic smokers were 1.4 times as likely as Whites to be CiST users. Conversely, African American, multiracial, and those reporting other race had lower odds of ST use compared to white female smokers. Female smokers having less than high school education were 1.2 times more likely to be CiST users compared to college graduates, whereas women with high school or some college education were less likely to be CiST users than college graduates. Compared to female smokers employed for wages, smokers who were out of work, students, or unable to work had increased likelihood of CiST use but self employed, retired, and homemaker female smokers had decreased odds of CiST use. CiST use was 1.3 times more likely among female smokers who were also heavy drinkers compared to those who consumed less than one drink per day.

#### 3.2.2. Cigarette Smoking among Female ST Users

Among female ST users, 42.4% also smoked cigarettes. Multivariate logistic regression analysis showed that White female ST users were more likely to be CiST users compared to ST users of any other racial ethnic group and were 7.1 times as likely as African American female ST users to report CiST use. Female ST users who had less than high school education were also less likely to be CiST users compared to college graduates. However, high school graduates or those with some college education were more likely to be CiST users than college graduates. Similarly, female ST users who were self-employed, out of work, homemaker, students, or unable to work had increased odds of CiST use compared to female ST users who were employed for wages. Women using ST and having household incomes between $10,000 and 49,999 had higher odds of CiST use compared to female ST users with incomes less than $10,000. Conversely, female ST users earning more than $50,000 were less likely to use CiST compared to those earning less than $10,000.

## 4. Discussion

Previous studies have used a variety of terms to refer to the use of multiple forms of tobacco, and some terms had multiple meanings in the literature, so Klesges and colleagues called for common operational definitions but did not offer specific definitions [20]. In the present study, we have introduced “CiST” and defined it as the combined use of cigarettes and smokeless tobacco at any frequency to differentiate it from other concurrent tobacco use. We examined CiST use among males and females and identified sociodemographic factors associated with CiST use. Comparisons were made between CiST users and nontobacco users, exclusive smokers and exclusive ST users separately. This is the first study to evaluate CiST use patterns among females and factors associated with CiST using these three comparison groups.

Some characteristics of CiST users identified in the current study were similar to those found in previous research, such as a higher prevalence of CiST use in younger age groups compared to smokers and ST users [10, 20]. Likewise, our findings that lower education levels are associated with CiST use are consistent with previous work reported by the two studies conducted among military groups [20, 21]. However, our study found a stronger relationship between education and CiST use compared to nontobacco users for both genders, and the strength of this relationship is stronger among men than among women. Further, the association we found between alcohol consumption and CiST use among smokers is comparable to that reported by Klesges among Air Force recruits [20]. Although Spangler and colleagues described CiST use among the Lumbee tribe in North Carolina in 2001 [11], there are no other studies we are aware of describing CiST use among Native Americans. Our findings provide a national perspective regarding CiST use among Native Americans and come at an important time as many Native American tribes are developing tobacco control programs in their communities, and these findings suggest CiST use should be monitored among Native Americans.

Approaches used by previous researchers to compare concurrent tobacco use have been inconsistent and insufficient. Most previous work investigated ST use among smokers to better understand CiST use in this smoking subgroup [4, 10, 20, 21]. These analyses can be helpful in identifying groups of smokers at higher risk of CiST use; however, other information may be lost if this is the only comparison group used. Demographic and other factors related to CiST use may also be related to smoking, so using smokers as the comparison group may distort the relationships between CiST use and those factors. For example, this study found that CiST use was more prevalent among Native American men (2.8%) compared to White men (1.9%); however, when CiST use was evaluated among male smokers, the odds of CiST use was lower among Native Americans than Whites. In contrast, when nontobacco users were the comparison group, the odds of CiST use among Native American men was higher than White men. Comparing CiST users to nontobacco users provides information regarding factors associated with CiST use without distortion of the relationship. On the other hand, examining CiST use among tobacco using subgroups (smokers or ST users) offers insight regarding tobacco users who may be at higher risk of CiST use within the respective tobacco using group. With the recent tobacco industry marketing of smokeless tobacco to cigarette smokers [5], it is indeed important to understand groups of smokers who may be at risk for CiST use. Nevertheless, it may be equally important to understand other avenues of initiation to CiST use since information is currently lacking regarding how CiST use develops.

In addition to considering that CiST use may begin when monotobacco users adopt the other tobacco product, we need to consider another possible path to CiST use: the initiation of both forms of tobacco simultaneously. More information is needed regarding the development of CiST use among smokers, smokeless tobacco users, and in general. Until more is known about the development of CiST use, we recommend using more than one comparison group for surveillance of CiST use to enable a comprehensive examination of trends in CiST use.

Another important feature of the present study is the stratified analysis by gender. Although most of this study's findings for male tobacco users agree with past studies, this study also identifies sociodemographic characteristics associated with CiST use among women. A few previous studies of concurrent tobacco use have included women in their analyses [10, 19–21]; however, none examined CiST use among women separately. Even though the prevalence of CiST use is less than one percent of the female population, based on the results of current study, an estimated 500,000 women in USA are CiST users. Our findings show that certain groups of women are more likely to be CiST users, including AI/AN women, those with lower education, out of work, and heavy drinkers. In addition, CiST use is an emerging public health problem and its use among women may increase in the future since tobacco companies are marketing smokeless tobacco to smokers when smoking is restricted [5].

Using BRFSS data enabled the evaluation of CiST use patterns among males and females and the use of multiple comparison groups, with sufficient sample size and adequate power for the statistical analyses. Additionally, these data provide valid national estimates and the results are more generalizable to the US population. Unlike past studies, the large sample also enabled evaluation of more detailed categories within each sociodemographic factor, and a number of important categories were identified.

There are a few limitations of this study which are primarily inherent to BRFSS. The tobacco use prevalence estimates reported in current study are less than the estimates based on 2010 National Health Interview Survey (NHIS). BRFSS is a telephone-based survey that does not include households without landline phones, and this limitation of the sampling frame of the survey results in noncoverage bias. Similarly, estimates obtained from BRFSS are potentially biased due to low response rates which are associated with underrepresentation of certain subgroups of population such as, women, racial/ethnic minorities, younger adults, and low-income individuals [28]. These limitations may make it more difficult to estimate tobacco use in these underrepresented groups. Some previous studies have reported significant relationship between CiST use and tobacco use characteristics, such as age at smoking initiation, number of days (per month) of tobacco use, and quantity used per day; [20, 21] however, BRFSS lacks this information so these characteristics could not be examined. Validity of self-reported cigarette smoking has been assessed using biochemical specimens in the past; however, there are no such validation studies for ST use [29, 30]. There may be some misclassification bias due to self-reported tobacco use in the current study. Finally, BRFSS did not collect information regarding other forms of tobacco, such as pipes, cigars, bidis, or hookahs. Therefore, the nontobacco users category may include some users of these forms of tobacco.

## 5. Conclusions

This study identified a number of sociodemographic characteristics associated with CiST use and differences in these associations among women and men by factors such as employment status, educational attainment, and race. Hence, CiST use should be monitored and studied further in women and the high-risk groups in both genders identified in this study. This study also provided more detailed information of CiST use in specific categories not well studied previously, such as AI/AN, and various employment and income categories. Future monitoring of CiST use should continue to determine if CiST use changes over time, especially among high-risk groups. Finally, this study highlights the need to carefully consider what comparison groups should be used to examine factors associated with CiST use. Since information is currently lacking regarding how CiST use develops, and associations of CiST use vary with different comparison groups, tobacco surveillance systems should monitor a wide range of tobacco consumption and researchers should cautiously select comparison groups that are most appropriate for their investigation.

## Figures and Tables

**Table 1:** Sociodemographic characteristics of CiST users by gender—BRFSS 2010.

Variable	Male (weighted %)	Female (weighted %)
Age		
18–24	22.19	14.11
25–34	32.45	19.33
35–44	18.96	18.96
45–54	14.52	22.35
55–64	8.00	13.81
65 or older	3.88	11.43
Race ethnicity		
White	79.52	72.93
African American	5.76	9.26
American Indian/Alaska Native	2.22	3.67
Hispanic	7.07	11.09
Multiracial	2.85	2.41
Other	2.57	0.65
Education		
Less than high school	18.08	19.77
High school	43.69	37.22
Some college	25.38	26.60
College graduate or more	12.85	16.41
Occupation		
Employed for wages	53.67	38.37
Self-employed	10.61	3.87
Out of work	17.98	14.40
Homemaker	0.23	9.88
Student	5.47	5.12
Retired	4.40	9.31
Unable to work	7.64	19.06
Income		
Less than $10,000	7.16	14.90
$10,000–$14,999	6.05	10.65
$15,000–$19,999	10.44	14.96
$20,000–$24,999	12.12	12.49
$25,000–$34,999	15.14	12.21
$35,000–$49,999	13.53	10.41
$50,000–$74,999	15.35	6.60
$75,000 or more	20.21	17.79
Marital status*		
Married	67.94	62.26
Single	32.06	37.74
Alcohol drinking		
Light, moderate, or no drinking	84.69	88.78
Heavy	15.31	11.22

*Married: married or member of an unmarried couple; Single: divorced, widowed, separated, and never married.

**Table 2:** Prevalence of tobacco use by sociodemographic characteristics among males—BRFSS 2010.

Variable	Unweighted sample size	CiST user (weighted %)	Exclusive smoker (weighted %)	Exclusive ST user (weighted %)
Age				
18–24	5795	3.17	19.25	4.55
25–34	12675	3.10	24.13	4.73
35–44	22437	1.45	16.87	6.03
45–54	33851	1.19	18.98	3.88
55–64	41306	0.86	16.68	2.66
65 or older	54050	0.40	8.42	2.54
Race ethnicity				
White	134692	1.86	16.64	5.20
African American	11049	1.02	22.39	1.85
American Indian/Alaska Native	2515	2.78	31.08	6.69
Hispanic	11382	0.81	18.03	1.20
Multiracial	3024	2.64	27.17	5.45
Other	4642	0.85	11.70	1.63
Education				
Less than high school	15951	2.86	29.10	3.92
High school	48900	2.50	24.51	5.43
Some college	41238	1.69	18.91	4.81
College graduate or more	63389	0.56	7.89	2.85
Occupation				
Employed for wages	71785	1.63	15.66	4.83
Self-employed	20369	1.58	17.15	4.21
Out of work	11589	2.95	32.94	3.32
Homemaker	456	0.97	30.36	4.41
Student	2629	1.80	11.61	3.85
Retired	51632	0.44	10.27	2.55
Unable to work	10827	2.43	34.24	4.48
Income				
Less than $10,000	6398	2.56	32.54	3.07
$10,000–$14,999	7569	2.20	29.66	3.41
$15,000–$19,999	10224	2.57	28.72	3.43
$20,000–$24,999	13557	2.34	25.24	4.31
$25,000–$34,999	17385	2.43	21.98	4.30
$35,000–$49,999	23607	1.61	18.35	4.46
$50,000–$74,999	25191	1.54	14.28	4.68
$75,000 or more	47655	0.89	9.81	4.25
Marital status				
Married	111209	1.22	13.75	4.34
Single	58178	2.43	25.31	3.82
Alcohol drinking				
Light, moderate, or no drinking	155113	1.45	16.07	3.99
Heavy	8718	4.51	36.84	6.90

Total		1.62	17.45	4.16

**Table 3:** Association between sociodemographic factors and CiST use among males.

Variable	Nonuser OR (95% CI)	Exclusive smoker OR (95% CI)	Exclusive ST user OR (95% CI)
Age			
18–24	6.75 (6.67, 6.83)	3.14 (3.11, 3.18)	2.72 (2.68, 2.76)
25–34	10.54 (10.43, 10.66)	2.44 (2.42, 2.47)	4.00 (3.94, 4.05)
35–44	4.50 (4.45, 4.55)	1.38 (1.36, 1.39)	1.49 (1.47, 1.51)
45–54	3.19 (3.15, 3.23)	1.04 (1.03, 1.06)	1.96 (1.93, 1.98)
55–64	2.14 (2.12, 2.17)	0.86 (0.85, 0.87)	2.05 (2.02, 2.07)
65 or older	Referent		
Race/ethnicity			
White	Referent		
Af Am	0.28 (0.27, 0.28)	0.40 (0.40, 0.41)	1.21 (1.19, 1.22)
AI/AN	1.21 (1.19, 1.22)	0.78 (0.77, 0.79)	0.89 (0.88, 0.90)
Hispanic	0.15 (0.15, 0.15)	0.32 (0.32, 0.32)	1.71 (1.69, 1.72)
Multiracial	1.23 (1.22, 1.24)	0.81 (0.80, 0.82)	1.19 (1.17, 1.20)
Other	0.43 (0.43, 0.43)	0.62 (0.62, 0.63)	1.89 (1.87, 1.92)
Education			
Less than high school	7.53 (7.48, 7.58)	1.50 (1.49, 1.51)	2.52 (2.50, 2.54)
High school	4.39 (4.37, 4.42)	1.30 (1.29, 1.31)	1.60 (1.59, 1.61)
Some college	2.92 (2.91, 2.94)	1.19 (1.18, 1.20)	1.33 (1.32, 1.33)
College graduate or more	Referent		
Occupation			
Employed for wages	Referent		
Self-employed	0.99 (0.98, 1.00)	0.92 (0.91, 0.92)	0.91 (0.91, 0.92)
Out of work	1.20 (1.20, 1.21)	0.74 (0.74, 0.74)	1.50 (1.49, 1.51)
Homemaker	0.65 (0.63, 0.67)	0.39 (0.38, 0.40)	1.02 (0.99, 1.06)
Student	0.45 (0.44, 0.45)	0.99 (0.98, 0.99)	0.81 (0.80, 0.82)
Retired	0.59 (0.59, 0.60)	0.66 (0.65, 0.66)	0.72 (0.71, 0.73)
Unable to work	1.35 (1.34, 1.36)	0.96 (0.95, 0.96)	1.10 (1.09, 1.11)
Income			
Less than $10,000	Referent		
$10,000–$14,999	0.84 (0.83, 0.85)	0.90 (0.89, 0.90)	0.76 (0.75, 0.77)
$15,000–$19,999	1.04 (1.04, 1.05)	1.05 (1.05, 1.06)	0.99 (0.98, 1.01)
$20,000–$24,999	0.99 (0.99, 1.00)	1.01 (1.00, 1.02)	0.80 (0.79, 0.81)
$25,000–$34,999	0.99 (0.98, 1.00)	1.08 (1.07, 1.09)	0.90 (0.89, 0.91)
$35,000–$49,999	0.65 (0.65, 0.66)	0.91 (0.90, 0.92)	0.62 (0.62, 0.63)
$50,000–$74,999	0.61 (0.60, 0.61)	1.11 (1.10, 1.12)	0.63 (0.62, 0.63)
$75,000 or more	0.44 (0.44, 0.44)	0.94 (0.93, 0.95)	0.46 (0.46, 0.47)
Marital status			
Married	Referent		
Single	1.41 (1.41, 1.42)	0.93 (0.93, 0.94)	1.67 (1.66, 1.68)
Alcohol consumption			
Nondrinker, light, or moderate	Referent		
Heavy	4.26 (4.24, 4.28)	1.16 (1.15, 1.16)	1.69 (1.68, 1.70)

Odds ratios are adjusted for all other variables under study.

**Table 4:** Prevalence of tobacco use by sociodemographic characteristics among females—BRFSS 2010.

Variable	Unweighted sample size	CiST user (weighted %)	Exclusive smoker (weighted %)	Exclusive ST user (weighted %)
Age				
18–24	6826	0.56	14.72	0.63
25–34	22236	0.41	18.21	0.61
35–44	36206	0.33	15.64	0.38
45–54	52887	0.40	18.25	0.39
55–64	64731	0.31	14.83	0.38
65 or older	98075	0.20	7.67	0.53
Race ethnicity				
White	217259	0.36	16.00	0.33
African American	25166	0.30	15.41	1.16
American Indian/Alaska Native	3519	1.46	29.76	1.37
Hispanic	20528	0.28	9.18	0.46
Multiracial	4664	0.50	22.90	0.37
Other	6264	0.06	5.12	0.97
Education				
Less than high school	27344	0.68	21.14	1.26
High school	85570	0.47	20.08	0.59
Some college	78152	0.33	17.38	0.34
College graduate or more	88921	0.16	6.84	0.25
Occupation				
Employed for wages	107128	0.30	14.57	0.42
Self employed	16743	0.23	14.16	0.38
Out of work	15543	0.67	26.22	0.59
Homemaker	33692	0.23	11.88	0.43
Student	4506	0.38	11.92	0.30
Retired	80711	0.19	8.29	0.46
Unable to work	21212	1.11	31.00	0.95
Income				
Less than $10,000	15946	0.83	24.22	1.37
$10,000–$14,999	17242	0.67	22.90	0.56
$15,000–$19,999	21391	0.71	23.03	0.62
$20,000–$24,999	25277	0.48	20.02	0.54
$25,000–$34,999	29411	0.41	17.70	0.43
$35,000–$49,999	34984	0.27	16.40	0.26
$50,000–$74,999	35304	0.16	12.92	0.30
$75,000 or more	55437	0.21	8.16	0.25
Marital status				
Married	147693	0.28	12.43	0.39
Single	131848	0.45	18.75	0.60
Alcohol drinking				
Light, moderate, or no drinking	262219	0.31	13.95	0.47
Heavy	11298	0.89	31.10	0.43

Total		0.35	14.79	0.47

**Table 5:** Association between sociodemographic factors and CiST use among females.

Variable	Nonuser OR (95% CI)	Exclusive smoker OR (95% CI)	Exclusive ST user OR (95% CI)
Age			
18–24	3.12 (3.06, 3.18)	1.08 (1.06, 1.09)	1.06 (1.03, 1.09)
25–34	3.75 (3.69, 3.81)	0.76 (0.75, 0.77)	2.43 (2.38, 2.50)
35–44	3.31 (3.26, 3.37)	0.72 (0.71, 0.73)	2.56 (2.50, 2.62)
45–54	3.04 (2.99, 3.08)	0.68 (0.67, 0.69)	2.58 (2.52, 2.64)
55–64	1.90 (1.87, 1.93)	0.66 (0.65, 0.67)	1.84 (1.80, 1.88)
65 or older	Referent		
Race/ethnicity			
White	Referent		
Af Am	0.39 (0.39, 0.40)	0.76 (0.75, 0.77)	0.14 (0.14, 0.14)
AI/AN	1.82 (1.78, 1.86)	1.58 (1.55, 1.61)	0.35 (0.34, 0.36)
Hispanic	0.32 (0.31, 0.32)	1.40 (1.38, 1.41)	0.35 (0.35, 0.36)
Multiracial	1.08 (1.06, 1.11)	0.99 (0.97, 1.01)	0.60 (0.58, 0.62)
Other	0.15 (0.14, 0.16)	0.51 (0.49, 0.53)	0.05 (0.05, 0.05)
Education			
Less than high school	4.69 (4.62, 4.75)	1.18 (1.16, 1.19)	0.84 (0.83, 0.86)
High school	2.91 (2.88, 2.94)	0.95 (0.94, 0.96)	1.11 (1.09, 1.13)
Some college	1.87 (1.85, 1.89)	0.74 (0.73, 0.75)	1.38 (1.35, 1.40)
College graduate or more	Referent		
Occupation			
Employed for wages	Referent		
Self-employed	0.81 (0.80, 0.83)	0.86 (0.84, 0.87)	1.69 (1.65, 1.74)
Out of work	1.75 (1.73, 1.77)	1.19 (1.18, 1.21)	2.98 (2.92, 3.03)
Homemaker	0.70 (0.69, 0.71)	0.94 (0.92, 0.95)	1.11 (1.09, 1.13)
Student	0.76 (0.75, 0.78)	1.22 (1.20, 1.24)	2.01 (1.94, 2.08)
Retired	0.85 (0.83, 0.86)	0.94 (0.93, 0.96)	0.93 (0.91, 0.95)
Unable to work	2.99 (2.96, 3.02)	1.90 (1.88, 1.92)	2.55 (2.50, 2.59)
Income			
Less than $10,000	Referent		
$10,000–$14,999	0.89 (0.88, 0.90)	0.88 (0.87, 0.89)	2.75 (2.68, 2.81)
$15,000–$19,999	1.01 (1.00, 1.03)	0.99 (0.98, 1.00)	1.77 (1.73, 1.80)
$20,000–$24,999	0.63 (0.62, 0.64)	0.69 (0.68, 0.70)	1.14 (1.12, 1.16)
$25,000–$34,999	0.65 (0.64, 0.66)	0.81 (0.80, 0.82)	1.40 (1.38, 1.43)
$35,000–$49,999	0.43 (0.42, 0.44)	0.61 (0.60, 0.62)	1.34 (1.31, 1.37)
$50,000–$74,999	0.25 (0.24, 0.25)	0.47 (0.46, 0.48)	0.68 (0.66, 0.70)
$75,000 or more	0.32 (0.32, 0.33)	0.92 (0.91, 0.94)	0.95 (0.93, 0.97)
Marital status			
Married	Referent		
Single	1.23 (1.22, 1.24)	0.93 (0.92, 0.93)	1.49 (1.47, 1.51)
Alcohol consumption			
Nondrinker, light, or normal	Referent		
Heavy	4.44 (4.39, 4.49)	1.27 (1.26, 1.28)	3.23 (3.16, 3.30)

Odds ratios are adjusted for all other variables under study.
